# Prehabilitation in Alcohol Dependence as a Treatment Model for Sustainable Outcomes. A Narrative Review of Literature on the Risks Associated With Detoxification, From Animal Models to Human Translational Research

**DOI:** 10.3389/fpsyt.2019.00339

**Published:** 2019-05-16

**Authors:** Christos Kouimtsidis, Theodora Duka, Emily Palmer, Anne Lingford-Hughes

**Affiliations:** ^1^Centre for Psychiatry, Imperial College London, London, United Kingdom; ^2^Sussex Addiction Research and Intervention Centre (SARIC), School of Psychology, University of Sussex, Brighton, United Kingdom

**Keywords:** alcohol dependence, prehabilitation, withdrawal, detoxification, animal models, human research

## Abstract

In this review paper, we discuss how the overarching concept of prehabilitation is applicable to alcohol dependence. Central to prehabilitation are the concepts of expected harm, risks, and proactive planning to eliminate the harm or cope with the risks. We review the evidence from animal models, psychological experimental studies, as well as pharmacological studies, on the potential risks and harms associated with medically assisted alcohol detoxification and the current treatment paradigm for alcohol dependence. Animal models provide an approximation mostly of the physical aspect of alcohol withdrawal and detoxification process and make predictions about the development of the phenomena in humans. Despite their limitations, these models provide good evidence that withdrawal from chronic ethanol use induces cognitive impairment, which is worsened by repeated bouts of withdrawal and that these impairments are dependent on the duration of alcohol withdrawal. Initial clinical observations with alcohol-dependent patients confirmed increased incidence of seizures. In recent years, accumulating evidence suggests that patients who have had repeated episodes of withdrawal also show changes in their affect, increased craving, as well as significant deterioration of cognitive abilities, when compared to patients with fewer withdrawals. Alcohol dependence is associated with tolerance and withdrawal, with neuroadaptations in γ-Aminobutyric Acid-A Receptor (GABA-A) and glutamatergic *N*-methyl-D-aspartate (NMDA) receptors playing key roles. It is suggested that dysregulation of the NMDA receptor system underpins alcohol-related memory impairments. Finally, we discuss the Structured Preparation for Alcohol Detoxification (SPADe) as an example of how prehabilitation has been applied in clinical practice. We discuss the importance of partial control over drinking as an interim step toward abstinence and early introduction of lifestyle changes for both the patient and the immediate environment prior to detoxification and while the patient is still drinking.

## Introduction

The concept of pre-habilitation has been introduced in the field of orthopedics and describes a set of exercises and training routines for certain groups of patients with the aim to maximize physical strength and reduce the risk of expected harm or frequent injuries, therefore taking a proactive rather than a reactive approach. The concept is applied in surgery with the aim of preparing patients for a surgical intervention. It is a strategy for proactive management of risk factors associated with the surgical intervention. The approach is therefore described as a shift away from an impairment-driven reactive model and as an opportunity for introducing proactive sustainable and appropriate lifestyle changes (1).

Central to the successful implementation of pre-habilitation are the concepts of expected harm or risk and proactive planning. Both concepts are considered to be crucial determinants of the interaction between humans and the environment in general, associated with human evolution and the progress from hunting to agriculture, structured communities, and human civilization. Planning is crucial in all aspects of everyday life. The ability to predict or anticipate certain harm or assess certain risks is associated with the human ability of learning from experience, modify behavioral responses, and develop long-term and sustainable response strategies. To that effect, planning in advance of anticipation of risks can be considered as an essential strategy associated with individual survival and progress. Planning should not been viewed as a barrier for improvisation and innovation; on the contrary, it provides a stable environment for progress and positive change to take place.

The term “alcohol dependence” was first introduced in 1976 (2) and was used in both International Classification of Diseases (ICD-10) classification systems (3) and the *Diagnostic and Statistical Manual of Mental Disorders, Fourth Edition* (*DSM-IV*) (4). In *DSM-5*, dependence is now conceptualized on a continuum with abuse, such that a single disorder is now called alcohol use disorder (AUD) with mild, moderate, and severe sub-classifications (5). Alcohol withdrawal syndrome is a collection of symptoms that occur after an alcohol-dependent individual stops consumption (6). Withdrawal from alcohol has been associated with cognitive impairments in recovering alcohol-dependent patients and furthermore the risk of relapse after withdrawal is associated with cognitive deficit (7, 8).

In this paper, we introduce the concept of pre-habilitation and its role in the clinical management of alcohol dependence. The approach has many aspects that overlap with other clinical management interventions, such as harm reduction and opioid substitution treatment. The overall aim is to reduce medical and other associated risks in a safe environment and to empower the individual to achieve the psychosocial changes required for recovery and social reintegration. Here, we focus on alcohol detoxification and withdrawal, given that it poses substantial risks to cognitive function. We review the evidence from animal studies, human psychological experimental studies, and imaging studies. We have conducted a narrative review of preclinical and clinical evidence regarding alcohol withdrawal or detoxification using online resources, e.g., PubMed and Google Scholar, and that were published in English prior to September 2018. For the preclinical evidence, we have focused the review on studies using cognitive behavioral paradigms rather than those on physical withdrawal symptoms, e.g., seizures. For the clinical review, we have focused on neuroimaging studies of relevant neurobiological processes. We also discuss the limitations of current pharmacological interventions. Finally, we discuss, in some detail, an example of a clinical implementation of the model. In this paper, we have chosen to use the older and longer established term “patient” rather than client or service user. This choice does not refer to a scientific or philosophical position.

## Currently Recommended Treatment Paradigm to Manage Alcohol Detoxification

Current clinical guidelines suggest that medically assisted withdrawal or detoxification is generally required for the treatment of moderate to severe alcohol dependence. This should be planned and the importance of providing structured aftercare is emphasized (9). Medically assisted detoxification is required to minimize the risks of withdrawal-related symptoms and complications.

The guidelines suggest that the patient prepares for detoxification by attending sessions at a specialist service to enhance and maintain motivation to change and develop a plan for structured aftercare (9). As described, the latter is considered important to ensure effective treatment. What is delivered however may vary widely with sessions not necessarily providing structured preparation to address issues such as stabilizing the amount of drinking, enhancing partial control over drinking, promoting early lifestyle changes, or empowering changes within the immediate family or social environment.

Detoxification may be medically assisted as an outpatient in the community or as an inpatient in a general hospital or a specialist unit. The choice between these two detoxification settings depends on health risk factors and the availability of social support to mitigate these risk factors during the detoxification process, and it is usually made by the health professionals (9). Medically assisted detoxification is discussed in more detail in Section 6 below.

Structured aftercare (also referred to as rehabilitation) is considered by clinical guidelines as the most important component of the current treatment paradigm, with strong evidence for its effectiveness (9). It is recommended that the structured aftercare that follows detoxification be delivered within a Cognitive Behavioral Therapy approach, either on an individual basis or *via* membership of a Relapse Prevention Group, alongside family interventions. It is highly recommended that patients engage with peer-support or mutual aid groups, such as Alcoholics Anonymous or SMART Recovery. Pharmacological interventions such as acamprosate, naltrexone, or disulfiram are also recommended. The existing evidence does not favor outpatient over inpatient detoxification, or residential aftercare treatment over community treatment, or longer versus shorter duration residential aftercare treatment programs (9). However, access to residential aftercare programs is recommended for homeless individuals, and efforts should be made to address accommodation issues prior to discharge (9).

Two of the long-term challenges for professionals (both academics and clinicians) involved in the treatment of alcohol dependence are the definition of successful outcome as well as the high relapse rate. For example, statistics from Public Health England for the period 2017–2018 suggest that 61% of clients complete treatment successfully (i.e., are free from dependence, which could mean abstinence but not necessarily), the same proportion as the previous year (10). This number provides an indication of how successful treatment is but is dependent on the definition of successful treatment, the severity of presenting problem, and the time when completion and exit are reported. Other indicators such as maintenance of abstinence for 6 months and 12 months for alcohol-dependent patients could enhance our understanding of the effectiveness of the current treatment paradigm. Our local data suggest that only 60% of patients who have completed planned detoxification have been engaged in aftercare interventions, which are considered to be essential for long-term recovery (11). This ratio has improved to 82% when a pre-habilitation approach has been implemented, such as participation in the Abstinence Preparation Group (see Section 7 below) (12). There may be several explanations for this improvement, including benefits of participating in a group or more specific theory-based factors such as regaining of partial control over drinking and early lifestyle changes (12).

In summary, current treatment guidelines advocate avoidance of unplanned and urgent detoxifications as they do not lead to sustainable outcomes with regard to drinking behaviors (9). They put emphasis on the provision of psychological treatment following detoxification and promotion of participation in peer-support interventions (9). Given the challenges in improving treatment outcomes, we consider that the main shortfalls of these guidelines are that <br/>(1) the only therapeutic input prior to detoxification is restricted to motivation enhancement and preparation of an aftercare plan without any theory-based structured intervention to manage the risks expected once alcohol is withdrawn, and (2) a large proportion of patients completing detoxification do not engage with any evidence-based aftercare to reduce the risk for relapse. Given that the majority of psychological interventions may not have an immediate effect, and the high risk of relapse during the first 3 months post-detoxification, we need to consider an alternative approach such as pre-habilitation to reduce the risk of relapse. Furthermore, the fact that these interventions are taking place during a period of mood dysregulation, which is the result of the detoxification itself, might compromise their effect.

## Learning and Habit Development in Humans

Humans have the ability to test out a new behavior as a solution to a challenge and—depending on the results (e.g., reward)—to either consolidate or abandon this behavior. Consolidated rewarding behaviors then become repeated in similar (or different) situations and, over time, become automatized. This leads to the fast replication of such behaviors—a bypassing of the conscious and careful consideration of pros and cons—since the analysis of their efficacy has already been done, in the past, and proven successful (13). The ability to automatize successful behaviors allows humans to continue with further learning and the accumulation of new skills and expertise. This ability to bypass the conscious decision-making control mechanism confers the advantage of fast and successful responses to dangerous environmental stimuli, but it has a major disadvantage: humans are not able to monitor the appropriateness of the behavior or assess the possible need for behavioral modification (13).

Whenever an automatized behavior requires modification, the learning process must be slowed down, in order to allow for the decision-making process to again become conscious. This does not refer to a meta-cognitive process, but rather to the creation of time and space between the high-risk situation and the behavioral response. In other words, implicit cognitions must again become explicit if the individual is to regain conscious control in order to modify the extant behavior. It is easier to undertake this reversal process (14) in a safe, practice-friendly environment, where those factors necessitating the fast reproduction of a behavioral response may be kept under control. Factors such as stress, threat, or uncomfortable physical symptoms typically provoke instinctive responses of a habitual nature. Humans tend to think more clearly and laterally when they can explore alternative solutions without facing immediate threat or being subject to stress.

### The Expected Risk in Alcohol Dependence

In the case of drinking (as well as other substance misuse), this leads to the state whereby habitual drinking dominates all other behaviors and becomes repeated despite the person’s awareness of its loss of effectiveness and the accumulation of evidence of the associated harm. This leads the person into the paradox of wanting (implicit activation of need) although not liking (conscious desire and choice) drinking (15). From a psychological perspective, all explicit cognitions—such as positive and negative expectancies of the effect of drinking—which were conscious and under the control of the individual, are rendered implicit, and bypass the conscious decision-making pathway fuelling the continuation of drinking (13). This phenomenon is described as “loss of control”, an underlying theme common to 9 out of the 11 criteria of Alcohol Use Disorder in *DSM-5* (5), three out of six criteria for alcohol dependence in ICD 10 (3), and one of three in ICD 11.

In the sections below, we discuss the risks associated with alcohol withdrawal and medication-assisted detoxification interventions. We review the evidence from animal models, pharmacological studies, and psychological experimental studies to explore risks such as cognitive impairment, stress sensitivity, the limitations of medication-based protective roles, as well as limitations of the existing treatment paradigm of planned detoxification and rehabilitation.

## Animal Models of Alcohol Withdrawal and Detoxification on Cognitive Impact

Animal models have been used to try and understand the phenomenon of alcohol withdrawal and specifically to determine if repeated withdrawals particularly have an impact on cognitive function. Animal models have several advantages in alcohol research. They may be used to study determinants of alcohol-related behavior where there are ethical issues with carrying out such research in humans due to risks in giving volunteers or patients addictive harmful substances (16).

Further, animal models are used because animals have similar genetic, biochemical, and physiological compositions to humans. Therefore, research using animals can inform the understanding of the human condition and help lead to the development of new therapeutics. Some of the current medications approved for the treatment of alcohol use disorders (e.g., naltrexone and acamprosate) were developed using animal models (16). However, animal models do not represent the entire complex disorder; instead, they allow the study of component features of the condition and help provide evidence for the determinants of such behaviors (17).

There are currently several different methods used to model ethanol (alcohol) dependence in rodents such as forced consumption in drinking water, ethanol containing liquid diet, ethanol vapor inhalation, and repeated intraperitoneal or intragastric administration (18). In addition to route of administration, the length of ethanol exposure varies between models of alcohol use, e.g., from a 4-day chronic intermittent exposure (19) or a 6-month chronic model (20). The variation in both administration and duration of chronic ethanol administration complicates the interpretation of results. All of these models aim to mimic the neuroadaptations in the brain, which lead to tolerance and physical dependence of alcohol. A key issue with these models is the forced exposure to ethanol, which doesn’t accurately represent the compulsive element of the human experience of alcohol dependency despite efforts to assess operant re-enforcing and conditioned responses (16). Induction of alcohol dependency in animals is considered to be successful if withdrawal symptoms are present upon cessation of exposure (18). However, this is representative only of a physical dependency and lacks the complexity of all the environmental and psychosocial influences that contribute to the complex human experience of alcohol addiction.

Animal models of alcohol consumption have also been developed to reflect voluntary alcohol consumption such as the two-bottle choice test, using gradually increasing concentrations of ethanol or adding sweeteners (17). Although preference tests are often influenced mainly by taste, some animals show a preference for the pharmacological effects of alcohol, and this has allowed genetic manipulation to produce high or low alcohol preference breeds. Rodents will voluntarily consume up to 40% ethanol (16). For the study of alcohol withdrawal, these voluntary consumption paradigms are often not sufficient because consumption levels are not high enough to induce withdrawal symptoms. Another limitation of these procedures is the difficultly to determine an animal’s motivation to seek alcohol. Motivation to consume alcohol can be best demonstrated by an operant task model (such as lever pressing to receive alcohol in which the number of presses required increases) or a conditioned place preference task [for a detailed description, see (21)].

### The Impact of Withdrawal on Cognition

Physical withdrawal symptoms are similar in humans and animals and include tremors, agitation, rigidity, spontaneous seizures, audio sensitivity, handling-induced seizure sensitivity, and weight loss (22). However, alcohol withdrawal induces much more than just physical symptoms with low mood and anxiety evident. This negative affective state is thought to contribute to the risk of relapse in alcohol dependence and is therefore a critical area of study (these effects in humans are discussed in detail in Section 5 below). Withdrawal is thought to induce these effects *via* neuroadaptations from chronic ethanol’s exposure on brain areas that control fear and memory. For this review, we focused on the studies assessing the impact on withdrawal from chronic alcohol exposure on cognitive function in rodents, which are summarized in **Table 1**. This table shows evidence that cognitive deficits are seen in animal models of withdrawal, that this deficit can worsen with repeated withdrawal, and finally that this cognitive impact varies with the length of the withdrawal period.

**Table 1:** The effects of withdrawal on cognition; a summary of research using animal models.

Reference	Animal	Species	Gender	Age/weight	Chronic alcohol model	Daily alcohol intake	Withdrawal period	Cognitive testing/measure	Cognitive deficit present
(23)	Rat	Lister hooded	Male	250–300 g	Ethanol-containing diet for 24 days 2 × 3-day withdrawal episodes	13–14 g/kg	2 weeks 2 weeks 2 weeks + 1 month	Negative patterning task Contextual fear conditioning via a foot shock Spatial learning in the Barnes maze	YES NO NO
(24)	Rat	Sprague–Dawley	Male	160–180 g	In drinking water as sole source of fluid (20%) 8, 18, and 28 weeks	12.2 to 9.7g/kg	4 weeks	Eight arms radial maze (memory): Spatial Nonspatial	YES YES
(25)	Rat	Sprague–Dawley	Male	200–250 g	25% ethanol solution as the only source of fluid for 9 months (increasing concentrations from 10%)	53–26 mg%	2 weeks	Memory performance shuttle box: Active avoidance Passive avoidance	NO YES
(26)	Mouse	C57BL/6	Male	At least 70 days old	Chronic intermittent ethanol exposure ethanol vapor (16 h/day for 4 days with 8-h periods of withdrawal) 3 consecutive cycles, with 3 days of withdrawal IP primer 1.6 g/kg	175–225 mg/dl)	Up to 1 week	Behavioral attentional set-shifting task and reversal learning	YES
(27)	Rat	Wistar	Male	250–280 g	2 g/kg ethanol *via* gavage twice a day for 28 days	BAC up to 120 mg/dl	5 days	Open field (exploratory behavior)	YES (lower frequency of rearing)
(28)	Rat	Hooded Lister	Male	200–240 g	Nutritionally complete liquid diet 7% ethanol Single withdrawal, 24 consecutive days	17.5 g/kg BAC 100 mg/dl	12 days	Seizures (PTZ kindling) Conditioned emotional response	YES NO
					Nutritionally complete liquid diet 7% ethanol Repeated withdrawal, 30 days, with two periods of 3 days, 11, and 21, in which they received control diet		12 days	Seizures (PTZ kindling) Conditioned emotional response	YES (faster than either control or SWD rats) NO ↓
(19)	Rat	Sprague–Dawley	Male	275–325 g	Catheters in the stomach 5 g/kg 25% in diluted nutritionally complete diet Additional ethanol was administered every 8 h for 4 consecutive days	7.6 g/kg	4.5 days	Morris water maze (learning)	YES
(29)	Rat	Hooded Lister	Male	200–240 g	Nutritionally complete liquid diet 7% ethanol Single withdrawal, 24 consecutive days	12.5 ± 0.8 g/kg	2 weeks	Conditioned emotional response (fear conditioning low and high intensity): Suppression Extinction Reversal	NO NO NO
					Nutritionally complete liquid diet 7% ethanol Repeated withdrawal, 30 days, with two periods of 3 days, 11, and 21, in which they received control diet	12.9 ± 1.0 g/kg	2 weeks	Conditioned emotional response (fear low–high intensity): Suppression Extinction Reversal	NO YES YES
(30)	Rat	Hooded Lister	Male	200–240 g	Nutritionally complete liquid diet 7% ethanol Single withdrawal, 24 consecutive days	15.0 ± 0.4 g/kg	2 weeks	Pavlovian-to-instrumental transfer	YES
					Nutritionally complete liquid diet 7% ethanol Repeated withdrawal, 30 days, with two periods of 3 days, 11, and 21, in which they received control diet	15.3 ± 0.6 g/kg			YES
(31)	Mice	CD-1	Male	8 weeks	Only source of liquid increasing concentrations up to 20% ethanol 4 weeks	0.20 ± 0.01 g/dl	3 or 12 weeks after	T-Maze Foot shock Avoidance Greek Cross Brightness Discrimination Step-Down Passive Avoidance Shuttle box Active Avoidance	NO NO NO NO
					Only source of liquid increasing concentrations up to 20% ethanol 8 weeks			T-Maze Foot shock Avoidance Greek Cross Brightness Discrimination Step-Down Passive Avoidance Shuttle box Active Avoidance	YES YES YES YES
(32)	Mouse	C57BL/6J	Male		Vapor exposure 16 h with 8-h break	2.5–3.0 g/kg	32 h	Odor cue conditioning influence on voluntary ethanol consumption 15% presented in a free choice situation with water 5 days	YES
(33)	Rat	Wistar	Male	240–270 g	Nutritionally complete liquid diet as the sole source of nutrients. All rats received the CON liquid diet for an initial 3 days then (6% v/v) for 14 days	8.4–10.4 g/kg BEC 80.3 ± 7.5–132 ± 5.9 mg/dl	8 h, 48 h and 72 h	Elevated plus maze. (anxiogenic-like effect) Contextual fear conditioning (foot shock freezing)	YES (8 h only) YES
(34)	Rats	Lister hooded	Male	130–160 g 350–400 g	Nutritionally complete liquid diet 7% ethanol Single withdrawal, 24 consecutive days	18 g/kg Experiments 2 and 3 12.5 g/kg	8 h	Elevated plus maze (anxiety) Plasma corticosterone	YES YES ↑
					Nutritionally complete liquid diet 7% ethanol Repeated withdrawal, 30 days, with two periods of 3 days, 11, and 21, in which they received control diet			Elevated plus maze (anxiety) Plasma corticosterone	YES but no more than SWD NO
(35)	Mouse	C57BL6/J	Male	90–100 days 24–28 g	4 bouts of 16-h exposure to EtOH vapor separated by 8-h periods of withdrawal	64.76 ± 31.97 mg/dl	4 days continuous	Electrophysiological recording from surgically implanted electrode: Sleep time Sleep architecture	YES ↓ YES
(36)	Rat	Wistar	Male	250–300 g	Self-administration continuous (24 h/day for 7 days/week) or intermittent (24 h/day for 3 days/week) access to alcohol (20%) using a two-bottle choice procedure 5 months	<80 mg%	24 h to 68 days	Working memory performance in a Y-maze and the delayed nonmatching-to-sample task (DNMS) Elevated plus maze	YES (24–72 h) but not (16–68 days) NO
(37)	Mouse	Swiss	Male	8 weeks	1–8 weeks of intermittent access to 20% 3 days/week 24–48 h between EtOH access days, two-bottle choice	11.10–284.3 mg/dl	6–8 h	Aggressive and nonaggressive behaviors with a conspecific	YES (aggression and decreased social contact)
(20)	Mouse	C57/BL6	Male	4 months at start	Source of liquid, concentrated solutions of ethanol 4% the 1st week, 8% the 2nd week, and 12% for 6 months	15.34 ± 4.3 g/kg	Progressively withdrawn 1 week (1 W)	Working memory task. Spontaneous alternation was tested in a T-maze Elevated plus maze	YES YES
							6 weeks (6 W)	Working memory task. Spontaneous alternation was tested in a T-maze Elevated plus maze	YES NO
(38)	Mouse	C57BL/6J	Male	At least 10 weeks old	Chronic intermittent vapor inhalation. 16 h separated by 8-h periods of withdrawal <br/>ip primer dose	211.2 ± 25.0 208.8 ± 14.3 211.2 ± 25.0 208.8 ± 14.3	3 to 10 days	Social Approach Novelty-Suppressed Feeding Digging Bottle Brush Tests	NO YES ↑. YES ↑ YES ↑
		DBA/2J	Male	At least 10 weeks old	Chronic intermittent vapor inhalation. 16 h separated by 8-h periods of withdrawal <br/>ip primer dose	253.3 ± 16.0 173.3 ± 14.7 169.5 ± 19.9 179.5 ± 22.3	3 to 10 days	Social Approach Novelty-Suppressed Feeding Digging Bottle Brush Tests	YES NO YES ↑ YES ↑

### The Presence of Cognitive Impact

The experiments in **Table 1** used behavioral paradigms following a variety of chronic alcohol models to assess cognitive function including the elevated plus maze, the T maze, social interaction, and conditioned fear response learning. These have been used to demonstrate withdrawal-induced impairments in learning (19, 31), cognitive flexibility (26), memory (20, 24, 25, 31, 36), sociability (38), as well as increasing anxiety (23, 27) and sleep disruption (35). In addition to the previously described limitations associated with animal models of chronic alcohol consumption and withdrawal, these studies are also subject to the limitations of the behavioral paradigms used. For example, several studies that illustrate the effect of ethanol withdrawal on inducing anxiety in rodents use paradigms such as the elevated plus maze, the light–dark box, and the open field (18). Measures used in these paradigms such as line crossings or % of time spent in the center, can be influenced by impaired locomotion of the animal, as well as anxiety, and therefore these results may lack construct validity. However, taken together, given the multiple cognitive defects assessed, it can be concluded that alcohol withdrawal may induce some cognitive impairment.

### The Effect of Multiple Withdrawals

Several of the studies described in **Table 1** indicate the worsening of withdrawal symptoms given multiple withdrawal episodes, which is consistent with the clinical picture. The best documented example of this phenomenon in rodents is the frequency of seizures following several detoxifications: known as the kindling effect (39, 40). The kindling effect is defined by Pinel et al. as “the progressive intensification of elicited motor seizures that occurs during a series of convulsive stimulations”; this leads to increased susceptibility to convulsive seizures during alcohol withdrawal due to previous seizure-inducing withdrawals (39). The impact of multiple withdrawals also has a worsening effect on some of the associated cognitive impairments. This was demonstrated using rats fed an ethanol-containing diet (13–14 g/kg/day) for 24 days with two 3-day withdrawals compared with controls and with rats undergoing continuous ethanol treatment (23). These rats performed worse at negative patterning tasks but not spatial learning, which indicates that repeated withdrawals may affect some areas of cognition such as plasticity but not others. This differential effect of repeated withdrawals on only some cognitive defects is consistent with evidence that repeated withdrawals in rats compromised the acquisition of a conditioned fear response without impacting the recall of a previously learned fear association (29). These findings led to a hypothesis that multiple withdrawals induce aberrant neuronal plasticity, which gives rise to interesting predictions. Based on the idea that repeated withdrawal from alcohol results in repeated overactivity within glutamatergic systems (see below), it is possible that hyperactivation of glutamatergic systems would induce synaptic plasticity, leading to synaptic strength. If repeated withdrawals increase synaptic strengths, then stimulation of input pathways will have an enhanced effects on outputs, leading to certain excitability. However, if synapses are already strengthened, then the capacity for further plasticity will be reduced, leading to impaired learning of new associations (41, 42). However, further research is required to determine the underlying mechanism(s) behind multiple withdrawals reinforcing some but not all cognitive defects.

### The Duration of the Withdrawal Effect on Cognition

A key consideration is the duration of withdrawal from alcohol treatment. Some studies have looked at immediate effects of withdrawal after 8–24 h (23, 36, 37), while others assess cognitive defects present after a much longer period (several weeks) (25, 31). One key question is whether any cognitive impairment is long-lasting and/or persistent even following a significant period of abstinence. One study found that withdrawal caused significant working memory impairment during acute withdrawal (24–72 h) but not extended abstinence (16–68 days) (36). This contrasts with another study in mice in which short-term memory was not affected by withdrawal but learning and long-term memory were still impaired when tested 12 weeks after cessation of ethanol consumption (31). This suggests that withdrawal, while having a severe acute effect on cognition, may also cause long-lasting impairments. Therefore, the type of cognitive impairment present may also differ depending on the duration of abstinence.

#### Proposed Mechanisms of Withdrawal-Induced Cognitive Dysfunction

There is much discussion about the mechanism by which withdrawal from chronic ethanol induces cognitive impairments. Animal models have been used to link alcohol consumption with neurodegeneration and changing brain structure by neurotoxicity, reducing neurogenesis, and reducing the size of existing neurons. This has been related to dysfunctional behavior, which is suggestive of cognitive impairments (19). There have been several studies investigating the processes underlying these neurotoxicities. One such experiment in both rats and mice of both genders found increased levels of corticosterone in the brain tissue and plasma of both acutely (plasma) and prolonged (brain) withdrawn animals (43). Raised levels of corticosterone are known to cause neuronal damage, and it was therefore proposed as a potential mechanism underlying withdrawal-induced cognitive dysfunction. These raised corticosterone levels are thought to increase neuronal damage by potentiating excitatory transmission, inducing neuronal atrophy. Additionally, increased expression of NMDA receptors was found on the synaptic neurones of the medial prefrontal cortex, using a mouse model of chronic intermittent ethanol (26). This was also linked to a behavioral deficit in cognitive flexibility a week after the cessation of ethanol consumption. These findings suggest that the neuro-adaptive changes as a result of chronic alcohol consumption may contribute to withdrawal-induced dysfunction.

Other studies have focused on which brain areas are damaged during alcohol withdrawal, which may further inform how cognitive defects occur. For example, rat performance on a cognitive task was impaired by lesions of the basolateral amygdala (conditioned reinforcement and reinforcer devaluation) and central nucleus of the amygdala (Pavlovian-to-instrumental transfer) to identify which area is affected during single or repeated withdrawals. The result indicated that the central but not basolateral nucleus was affected during withdrawal. Similarity studies of mouse brains found that dendritic spine density was reduced in the lateral orbitofrontal cortex of mice following chronic intermittent exposure to ethanol (43). A comprehensive review of all relevant research is beyond the scope of this article; however, these examples provide evidence that the induction cognitive dysfunction following withdrawal is a complex process involving several brain regions. It is a vital area of research if we are to protect the brain, or at least limit the damage, in alcohol dependence.

#### Conclusion From Animal Models

Ultimately, there are several different animal models of chronic alcohol consumption that are used to study the impact of withdrawal on cognition. While these models fail to replicate all the complexities of psychosocial and compulsive factors that occur in the human experience of withdrawal, these animal models provide good evidence that withdrawal from chronic ethanol induces cognitive impairment, that this impairment is worsened by repeated bouts of withdrawal, and that these impairments are dependent on the duration of alcohol withdrawal and abstinence. These animal models have led to the identification of neuroadaptations and increased levels of corticosterone as potential modifiers of cognitive deficits caused by withdrawal and which brain regions are vulnerable to or involved in these impairments. Understanding the risks of withdrawal and the underlying neurobiology is vital if we are to develop more effective therapies for reducing the damaging consequences of alcohol withdrawal.

## Consequences of Repeated Detoxification of Patients Dependent on Alcohol

There is strong evidence that repeated detoxifications are associated with several cognitive and emotional impairments. Initial observations confirmed increased incidence of seizures (44–46). During recent years, accumulating evidence suggests that individuals who have experienced repeated episodes of withdrawal show changes to their affect, increased craving, as well as significant deterioration of cognitive abilities, when they are compared to patients with fewer withdrawals (47–49).

Several investigators had suggested that repeated episodes of detoxification increase the risk of withdrawal seizures. Further support to their suggestion came with the discovery of the differential response of alcohol-dependent patients to anxiety evoked by the noradrenergic alpha2 agonist, yohimbine, between those with two or more detoxifications compared to those with only one (50). These initial observations were followed by a plethora of experimental evidence showing that repeated experience of repeated detoxifications results not only in increased incidence of seizures and anxiety but also in increased craving and impaired inhibitory control of several behaviors in tasks (50, and in more detail below, e.g., 51, 52). Such tasks are challenging for high-order executive functions within problem solving or emotional evaluation contexts like reward seeking under conditions of incentive conflict, cognitive flexibility in an intra-extra dimensional shift, and reversal task and recognition of emotions in others.

Correspondingly, brain imaging shows that inaccurate performance on the cognitive tasks in alcohol dependence in humans who had experienced multiple detoxifications is associated with loss of gray matter in prefrontal regions; the loss of gray matter is positively correlated with the number of detoxifications. Evidence also suggests that the ability to recognize emotions in others (e.g., fearful faces) is associated with reduced connectivity between insula and prefrontal areas, but increased connectivity between insula and subcortical regions (colliculus) and between amygdala and other subcortical regions [e.g., bed nucleus of stria terminalis (BNST)].

Understanding the mechanisms that underlie the associations between repeated detoxifications and cognitive and emotional impairments as well as brain structure and functions alterations is mainly based on animal models [see previous section and (23)]. Additionally though, binge drinking (a tendency to drink excessively in one session leading to intoxication followed by abstinence) in young human adults has also been used as a model to explore possible predisposition to and early consequences of alcohol drinking in the form of repeated cycles (53–58).

Here, we will summarize the empirical evidence of the cognitive and behavioral deficits and their brain substrates associated with repeated detoxifications and how such deficits may increase vulnerability to relapse.

### Cognitive Control Processes Involved in Relapse

Increased urges to drink alcohol when induced by alcohol-associated stimuli and reduced ability to control the amount are recognized as the two basic processes of alcohol dependence. Inhibitory control is necessary for self-regulation. This is linked to executive function. Individuals who have low executive capacity or have damage to brain substrates subserving executive function display reduced ability for self-regulation and a greater susceptibility to behavior driven by stimulus and relapse (59, 60). Stimuli irrelevant to the present task or in contrast to the individual’s current goals can diminish self-regulatory behavior in a stimulus-driven fashion and lead to relapse (61, 62). Other evidence, however, suggests that a stimulus-driven effect may be dependent on search goals driven by the individual’s desire to consume alcohol (63). Several cognitive processes are considered to support self-regulation such as working memory and the ability to shift attention from previously relevant (but now irrelevant) stimuli (e.g., alcohol cues) to currently relevant factors (e.g., awareness of drinking consequences).

With the escalation of dependence, alcohol-associated stimuli become more salient and attract attention faster, thus diminishing the ability to inhibit the urge to drink. Such alcohol-associated attentional bias predicts relapse rates and treatment outcomes (64). Neuroimaging studies have provided strong evidence for the increased involvement of stimulus-driven networks (subcortical structures) and reduced involvement of brain substrates associated with cognitive control (65–67). Thus, as dependence progresses, relapse after several efforts to achieve and maintain abstinence becomes increasingly likely as distinct places, people, and paraphernalia associated with the reward offered by alcohol trigger an intense motivation within the addicted person to consume alcohol. As mentioned above, attentional processes (i.e., the ability to shift attention from previously relevant (but now irrelevant) stimuli to currently relevant factors may be crucial for self-regulation. Although impairments of cognitive control are associated with increased incidence of relapse in alcohol dependence, few studies have directly examined the possible impact of repeated detoxifications on cognitive control.

Alcohol-dependent individuals show impaired cognitive flexibility as measured in an intra–extra dimensional shift and reversal task (IED). This is associated with reduced volume of gray matter in a cluster within the inferior frontal gyrus (BA47) and the neighboring anterior insula. This is an area that shows reduced gray matter volume in alcohol-dependent patients and especially in those with a history of multiple detoxifications (52). The inferior frontal gyrus (IFG) is an area involved in inhibitory control. Observed decreased gray matter volume in this area suggests that decreased inhibitory control due to IFG damage may be linked with repeated relapses (68). Therefore, inhibitory control seems to modulate the translation of desire to drink into alcohol consumption and weakening of inhibitory control may lead to addiction (68). To that effect, strengthening inhibitory control may be an important cognitive strategy to prevent relapse (69).

### Social Competence as a Cause of Relapse: Brain Mechanisms

The cognitive deficits caused by reduced function of prefrontal brain areas (41, 42) in alcohol dependence, arising from repeated detoxifications, may not only contribute to inflexible behavior and perseveration of drinking but also to the impairments in social cognition, which is crucial for adaptive social interaction (70, 71).

Earlier studies have demonstrated that alcohol-dependent patients generally have reduced ability to recognize emotions expressed by facial expression in others (72–74). Our research has shown that such impairments may increase with greater numbers of detoxifications (75). Emotional recognition deficits are associated with less successful recovery (76, 77). A recent study that examined prospectively objective treatment outcomes found that alcohol-dependent patients who were poor in recognizing emotions in others were also more prone to relapse (78).

Neuroimaging findings have revealed brain changes associated with emotion recognition deficits most commonly in prefrontal cortex, amygdala, and insula brain areas (51, 52). The amygdala is the brain structure involved in processing of emotion (79) including the recognition of fearful facial expressions (80); the insula is associated not only with emotional processing but also with emotion regulation. Imaging the brain of alcohol-dependent patients during fear recognition in emotional facial expression of fear (74) revealed reduced connectivity between insula and prefrontal emotional regulatory regions (81–84). In particular, a reduced connectivity of insula with the anterior cingulate cortex (ACC), orbitofrontal cortex (OFC), and ventrolateral prefrontal cortex (VLPFC) was seen in alcohol-dependent patients with two or more detoxifications compared with either controls or patients with a single or no prior detoxification (51). Increased connectivity, also in patients with two or more detoxifications, was found between insula and a colliculus neuronal cluster, a region representing an important subcortical area for arousal mechanisms (85), as well as between amygdala and bed nucleus of stria terminalis (BNST). BNST has been identified as the key component area of stress-induced relapse in animal models of addiction (86). From these findings, it can be argued that increased connectivity in amygdala-related networks could lead to an increased emotional reactivity (84), whereas decreases in the network integrity of insula-related networks could lead to inappropriate analysis of the emotional input (87).

Importantly, the strength of connectivity between insula and areas involved in control of behavior and regulation of emotion (inferior frontal cortex, frontal pole) was negatively correlated with the number of detoxifications and with the ability to control drinking as evaluated by a self-rating questionnaire (ICQ; 51), suggesting a relationship between repeated detoxifications and the subjective perception of the ability to abstain. These findings further support that focusing treatment in reducing the impact of repeated experiences of detoxifications represents a reasonable approach.

### Incentive Conflict and Cognitive Control as a Cause of Relapse: Brain Mechanisms

From the above, it becomes clear that controlling drug taking depends on the ability of higher-level monitoring functions to interrupt the incentive process that is induced by the rewarding properties of the drug, but could also depend on the strengthening of the incentive process as addiction progresses (88).

Drug taking is considered as an impulsive choice for an immediate positive outcome based on previous hedonic experience or relief from pain or stress but on the possible expense of long-term health and social benefits. Alcohol dependence may impair processes that contribute to choice impulsivity (89), so that later consequences of drinking are not taken into account. For the alcohol-dependent patient trying for abstinence, the conflict between the desire to drink and the aim to abstain in order to avoid adverse consequences may be particularly strong, leading to erroneous choice at the time and a lapse.

We have studied aspects of the interaction between incentive learning and behavioral control using the incentive conflict task (ICT) (90). This is a version of negative patterning tasks used in cognitive psychology (91). When performing the ICT, subjects first learn that two independent discrete cues signal reward (money gains), and in this way, they acquire incentive properties. In a second phase, while the individual cues continue to signal reward, when presented together in a compound, they signal punishment (money losses). Participants have to learn to respond appropriately so that they respond to gain money when the stimuli are individually presented, but withhold responding to avoid money losses when the stimuli are presented in compound. The incentive conflict task is thus a task that puts demands on decision-making under conditions requiring conflict resolution. We have proposed that the task creates a conflict between abstaining and responding for reward, which is similar to that experienced by the patient before lapse. Therefore, the impaired ability of patients who have experienced multiple detoxifications to perform the task might reflect the consequences of the detoxification process itself on behavioral control.

As the number of previously experienced detoxifications increases, patients become increasingly impaired in withholding their responses in the condition of no reward, suggesting that the process of detoxification may engender brain changes that affect decision-making to avoid reward losses and lead to loss of control (90). This is consistent with deficits observed in a rodent version of the same task, in rats chronically exposed to alcohol (23). Importantly, in this well-controlled animal study, it was the number of withdrawal events (“detoxifications”) that determined the extent of the deficit.

Neuroimaging of the ICT task with human control participants shows activation of several areas but most importantly those of the supplementary motor area, striatum (including putamen), gyrus rectus, ventromedial prefrontal cortex (vmPFC), and superior frontal gyrus areas, which are implicated in cognitive and emotional processing of reward (91–93) and regulatory control over a behavioral response (94, 95). Smaller gray matter volume in alcohol-dependent patients in the areas where dysregulated brain responses are seen during ICT have been reported, such as vmPFC and superior frontal gyrus, even more so in patients who had experienced more detoxifications. This is consistent with suggestions that these smaller volumes are “brain damage” associated with the detoxification experience. Further, the smaller volumes likely are associated with impairments in motivational decision-making, which involves the vmPFC (96, 97), and behavioral control, which involves the superior frontal gyrus (94, 95). Activation changes of vmPFC is shared with the gambling task (97), which resembles incentive conflict in requiring decision-making. Alcohol-dependent patients with several detoxifications also show impairments in this task (98). These findings are further supported by a study (99) that found that resolution of emotional conflict was associated with activation of an area that included the vmPFC.

Blunted response of the vmPFC in alcohol-dependent humans to the presentation of stress cues, a condition that the ICT also possibly generates, has been found to predict the incidence of relapse (100). Higher incidence of relapse with the possibility of trying to detoxify again leads to experience of multiple detoxifications found in our studies to be associated with smaller gray matter volume in vmPFC. Aberrant responsiveness to vmPFC to stress (101) is proposed to be associated with autonomic neural system dysfunction probably induced by the decreased ability of vmPFC to regulate emotional responses to stress or conflict situations. Prefrontal gyrus activation on the other hand may be more associated with the attentional and executive processes involved in inhibitory control that govern responding to ICT (94, 95, 102). Recent work on brain network efficiency of patients with alcohol dependence has identified, among other areas, the superior frontal gyrus area to show reduced nodal efficiency, supporting reduced ability of this area to carry out its functional activity (103).

The damage induced by alcohol—and detoxification—is not restricted to the areas identified in the ICT experiments. For example, the inferior frontal gyrus has been implicated in previous research during cognitive set switching (104) and also when resolving decision conflict during an instrumental learning task (105). Again, decreased inhibitory control due to IFG damage may support the occurrence of repeated relapses.

## Brain Imaging of Alcohol Detoxification in Humans

Alcohol dependence is associated with tolerance and withdrawal with neuroadaptations in GABA-A and glutamatergic *N*-methyl-D-aspartate (NMDA) receptors playing key roles (106). Dysregulation of the NMDA receptor system is thought to underpin alcohol-related memory impairments (107).

### Imaging Glutamate in Humans

In humans, magnetic resonance spectroscopy (MRS) can be used to measure glutamate levels in the brain, albeit often with other metabolites and neurotransmitter and metabolic pools that cannot be robustly distinguished (108). A number of studies have reported greater glutamate levels in alcohol-dependent individuals during early withdrawal from alcohol.

One study reported greater MRS glutamate + glutamine (Glx) levels in the anterior cingulate cortex (ACC) at the start (day 1) of alcohol detoxification in alcohol-dependent individuals compared with controls, which normalized over the next 14 days (109). Benzodiazepines were used for treatment. Glx levels were not related to severity of alcohol withdrawal. Complementary preclinical translational studies showed that glutamate levels in the medial prefrontal cortex (mPFC) of ethanol-dependent rats were increased at 12 h of withdrawal compared with controls and during intoxication; the glutamate levels had declined by 60 h. A further study from the same group provided more evidence that a hyperglutamatergic state is associated with brain neurotoxicity. In both humans and rats, hippocampal glutamatergic function was found to be inversely related to volume, although notably, no differences were found with controls in either species (110). This may have been due to different methodology and lack of power to detect a group difference due to smaller hippocampal volume.

However, other studies have reported that human glutamate levels were lower in the ACC, dorsolateral prefrontal cortex (DLPFC), or parieto-occipital cortex (POC) 9 days after stopping drinking compared with “light drinkers” and normalized (i.e., increased) during the following month in ACC only (109). The authors suggested that their first time point may have missed the early elevation in glutamate reported by others and that, altogether, studies suggest that glutamate levels change during alcohol withdrawal and early abstinence. Although glutamate levels at the earlier time point were inversely associated with cognitive task performance, improved cognitive function was not related to any changes in glutamate or indeed other MRS markers [creatine, *N*-acetylaspartate (NAA), choline, and GABA]. Similarly, lower glutamine levels have been found in alcohol-dependent individuals who are still drinking, though breathalyzed negative at the time of the scan, compared with light drinkers (111). An inverse relationship between glutamate, but not glutamine, levels and number of heavy drinking days has been reported in ACC of alcohol-dependent participants but not light drinkers (18).

Higher levels of glutamate + glutamine in the nucleus accumbens and anterior cingulate have also been shown to be positively related to craving in recently detoxified alcohol-dependent individuals (112, 113). However higher levels have not always been reported in the anterior cingulate (112), which may suggest a differential rate of glutamatergic normalization in brain regions. No moderating effect of medication, e.g., diazepam or clomethiazole, was seen on glutamate levels and no relationship was seen with withdrawal symptoms (112). No cognitive measures were described in this study.

Although studies did not necessarily find any relationship of glutamate levels with clinical variables, this is likely due to the clinical heterogeneity of alcoholism in the small number of participants in these imaging studies. Due to the lack of appropriate longitudinal studies, it is not clear whether any differences in MRS-derived markers reflect the neurotoxicity or neuroadaptations from alcohol directly or predate alcohol consumption and increase the risk of an alcohol use disorder.

### Modulating Glutamatergic Function

In human alcohol-dependent individuals undergoing alcohol detoxification, those who received acamprosate compared with placebo resulted in a reduction in a glutamate:creatinine ratio between 4 and 25 days in the anterior cingulate (114). Diazepam was allowed if required during detoxification. It appears that any effect of acamprosate took a while to develop as it did not have an effect on alcohol withdrawal symptoms or on glutamate:creatinine ratio in the first few days of detox. Another study reported that glutamate levels were reduced after 4 weeks of acamprosate treatment compared with slight increases in those patients who did not receive acamprosate (113). The evidence from these studies is consistent with acamprosate having an “anti-glutamatergic” effect and that this likely underpins its clinical efficacy including reduction in craving. As no cognitive measures were obtained in the participants in either study, it is unclear if acamprosate did result in any cognitive benefits.

### Other MRS Markers

Other MRS markers of neuronal integrity and function have also been studied in alcohol use disorder. For example, evidence is not consistent with lower, higher, or no differences seen in the metabolite *N*-acetylaspartate (NAA), which is seen as a marker of neuronal integrity and function. This likely reflects the heterogeneity of the disorder and methodologies used. Nevertheless, there is evidence that NAA is lower as a result of heavy alcohol consumption, that it increases on stopping drinking, suggesting recovery, and that low thalamic NAA levels have been shown to be associated with poorer treatment outcomes at 3 months (115, 116).

### Imaging Inflammatory Response in Alcoholism

The inflammatory burden of alcohol consumption and dependence in regard to cognition is not well characterized in humans though it is likely to be an important target for treatment (115). Such inflammation may also contribute to alcoholism, increasing the risk of Alzheimer’s disease (117). Positron emission tomography (PET) imaging studies assessing microglial activity with translocator protein (TSPO) tracers have shown lower, rather than higher, availability in abstinent alcoholics (106, 118, 119). Indeed one study showed that TSPO binding was positively correlated with verbal memory performance (118). Therefore, these studies suggest that lower glial density or an altered activation state with lower TSPO expression may contribute to cognitive impairment in alcoholism.

### Treatment of Alcohol Withdrawal/Detoxification

As described, alcohol withdrawal and its complications develop as alcohol levels decrease and recurrent withdrawals result in increase in severity of symptoms due to kindling (120, 121). Such complications are also more likely in those alcohol-dependent patients who are hypoglycemic, hypokalemic, hypomagnesemic, or with infection or trauma (e.g., subdural hematoma) (120). Treatment of alcohol withdrawal generally attenuates the risk of such consequences, but too frequently, alcohol dependence is missed due to lack of appropriate questioning or disclosure, so appropriate treatment is not started. Clearly, since delirium tremens and seizures reflect brain toxicity, there may also be an effect on cognition; thus, their prevention is paramount to protect brain function and optimize recovery. The reader is directed to clinical guidelines concerning more information regarding treatment of alcohol detoxification and prevention of complications (9, 122, 123).

Medically assisted alcohol withdrawal is generally treated with a reducing regimen of a benzodiazepine (e.g., chlordiazepoxide, diazepam, and lorazepam) (120, 122, 123). An alternative regimen is “symptom-triggered”, where the benzodiazepine is given once symptoms meet a threshold for treatment. This requires regular monitoring of alcohol withdrawal symptoms with a validated scale [e.g., Clinical Institute Withdrawal Assessment for Alcohol (CIWA-Ar)] by appropriately trained staff and so is not suitable in all circumstances, e.g., a busy admissions unit or nonverbal patients. Other anticonvulsants may be used (e.g., carbamazepine and sodium valproate); however, a Cochrane review did not find evidence in favor of their use to treat alcohol withdrawal (124). It should be remembered that benzodiazepines are also effective anticonvulsants and therefore risk of alcohol-related seizures can be managed with sufficient doses rather than adding in another anticonvulsant (123).

Another important clinical intervention to reduce risk of brain toxicity is consideration of thiamine deficiency as this vitamin is a key co-factor in metabolism. Thiamine deficiency may present with “paresthesia” (pins and needles) in hands and feet with numbness and with Wernicke’s encephalopathy (WE), which is a medical emergency. Clinicians are advised to be suspicious as the classic triad of confusion, ataxia, and ophthalmoplegia, suggesting the diagnosis of WE, are rarely seen together, whereas the first two symptoms are very commonly seen in alcoholism (123, 125). Clinically, thiamine deficiency and WE are generally only considered with alcohol detoxification when greater metabolic load increases the risk; however, it may occur at any time and in other addictions with poor diet and absorption. For those with WE or at risk of it, parenteral thiamine is required since absorption from oral thiamine is insufficient to replenish stores (122, 123, 125). Thus, giving thiamine appropriately is a critical intervention to protect brain function and prevent irreversible alcohol brain-related brain disorder.

As described, current clinical treatment with benzodiazepines may not be optimal in attenuating the hyperglutamatergic state of alcohol withdrawal. As described, MRS studies have shown that acamprosate reduces glutamate in the brain. Clinically, acamprosate appears to be well tolerated during alcohol detoxification, when added to benzodiazepines, though there is no impact on alcohol withdrawal symptoms as measured with the CIWA-Ar (114, 126). However, acamprosate during alcohol detoxification has been noted to improve sleep and reduce arousal levels (alpha slow-wave index) when assessed with magnetoencephalography (127). Therefore, it is unclear if acamprosate-related reduction in glutamatergic activity does improve cognitive outcomes either in the short term or in the longer term.

## Examples of Implementation of Pre-Habilitation in Alcohol Dependence

As described, the concept of pre-habilitation can be applied to the treatment of alcohol dependence, such as our model: “Structured Preparation for Alcohol Detoxification” (SPADe). Although SPADe has been applied on an individual basis, primarily it has been applied as an open, rolling group program, and described initially as Preparation for Alcohol Detox (PAD) and more recently as Abstinence Preparation Group (APG). The intervention may be regarded as a modified Cognitive Behavioral Therapy approach (128, 129), which is offered prior to detoxification and while the person is still drinking. The basic components of this treatment approach include (i) partial control over drinking, (ii) introduction of lifestyle changes for the individual, (iii) and the immediate family and social environment. Existing evaluations of SPADe treatment pathways suggest that about 72% of individuals with alcohol dependence presenting for treatment can engage and complete the pre-habilitation intervention (APG) (12).

### Partial Controlled Drinking

When presented as an alternative to lifelong abstinence as the sole treatment outcome (130), the concept of controlled drinking generates intense conflict within the field of addiction medicine. However, within clinical guidelines (9) controlled drinking within “healthy” limits may be considered as an appropriate treatment objective for harmful drinkers. For dependent drinkers, abstinence remains the preferred treatment objective (9).

The main aim of pre-habilitation is to pre-empt clinical withdrawal symptoms and the associated urges to drink. Within the SPADe treatment approach for alcohol dependence, controlled drinking is referred to as “partial” for two reasons: (i) it is an intermediate treatment stage rather than the final treatment aim, which is abstinence; and (ii) the amount and pattern of drinking are not within healthy limits. Therefore, within SPADe, the primary aim of the “partial controlled drinking” stage is to stabilize both the amount of alcohol consumed and the pattern of drinking. Alcohol is considered as “if it were a medication” with frequent and regular dosing to prevent the onset rather than to treat the appearance of withdrawal symptoms. This proactive elimination of symptoms is considered fundamental from a biological perspective, since it protects against acute brain dysregulation, which, in turn, might sensitize the brain, leading to an exaggeration of the negative impact associated with the disturbance of the brain homeostatic system. From a psychological perspective, it empowers the individual by restoring some control over decision-making and reducing the impulsivity associated with the experience and avoidance of cravings and withdrawal symptoms. Furthermore, partial controlled drinking provides a relatively stable environment for the individual—and their social group–to begin implementing lifestyle changes that lead to an increased sense of self-efficacy. This is considered the final mediating factor in social learning theory and cognitive behavioral treatment models (131).

The aim is to avoid substantial and dramatic reductions to the amount of alcohol consumed, which not only will prove unsustainable but might also lead to the precipitation of withdrawal symptoms, which could be life threatening. Thus, small sustainable changes are implemented, and once stability is achieved, a further gradual reduction of alcohol intake can be safely undertaken. In our experience, about half of the patients following this approach will be able to come off alcohol without the use of detoxification medication (12). This model of detoxification is called “guided self-detox”, and alcohol is regarded as if it was a medication that is gradually discontinued.

### Early Introduction of Lifestyle Changes

The stabilization of drinking provides for a short period a relatively stable and safe environment for the patient, the immediate family, and the patient’s social network to develop and test out lifestyle changes. Such early and gradual changes implemented within the individual’s lifestyle are necessary to provide (i) a routine in everyday life that will protect against early relapse, (ii) a response to the void that alcohol detoxification would otherwise leave in its wake, (iii) a distraction strategy against the onset of craving, (iv) an enhancement of personal responsibility, (v) a de-mystification of alcohol and a challenge to the omnipotence of cravings or withdrawal symptoms, and, finally, (vi) protection against the acute sense of stress experienced in the early days of abstinence.

The involvement of family members and the immediate social support system in treatment helps in reframing the environment, modifying unrealistic expectations, and supports the gradual adaptation to the new family dynamics (following the removal of alcohol). It will help in managing the anxiety and difficult feelings/emotions associated with broken trust and promotes a partnership approach. Fundamentally, recovery is easier to achieve and more sustainable within a respectful, stress-free, and supportive environment. It is far easier for the patient to maintain abstinence (in particular during the first few weeks) within a family environment that is also abstinent, thus removing proximal cues/triggers (smell or sight of alcohol) as well more distant cues, such as elevated levels of stress or negative emotional states.

## Conclusion

In this review, we have described how alcohol detoxification is a neurobiologically challenging time for the brain and is associated with cognitive impairments that contribute to the high risk of relapse. Despite their limitations, animal models have demonstrated that alcohol withdrawals induce impairments in learning, cognitive flexibility, memory, sociability, increased levels of anxiety, and disrupting sleep. The evidence is mixed on the duration of these effects, suggesting that, potentially, in addition to the acute effects, there might be long-lasting impairments. Furthermore, repeated withdrawals may affect some areas of cognition such as plasticity but not all. Evidence supports roles for elevated levels of corticosterone or increased expression of NMDA receptors in neuro-adaptations underpinning alcohol withdrawal.

How does this evidence translate into human patients? There is evidence that with repeated detoxifications, withdrawal seizures, levels of anxiety, and experience of cravings increase, whereas inhibitory control of certain behaviors such as reward seeking, cognitive flexibility, and recognition of emotions in others is reduced. Furthermore, attentional bias towards alcohol-associated stimuli is increased and predicts relapse rates and poorer treatment outcomes.

The evidence from neuroimaging studies is unable to clarify whether any differences observed reflect the neurotoxicity or neuro-adaptations from alcohol directly or predate alcohol consumption and increase the risk of an alcohol use disorder. Nevertheless, it seems that current clinical treatment with benzodiazepines may not be optimal in attenuating the hyperglutamatergic state of alcohol withdrawal.

How could the above evidence guide our clinical practice? The evidence reviewed in this paper suggests that the process of detoxification from alcohol in humans seems to have a negative impact on cognitive functioning and create or worsen mood dysregulation. These effects are temporal, although the exact duration is not specific as multiple factors might have an effect beyond and above the severity of the baseline alcohol intake (chronicity, amount, and pattern). Nevertheless, given that this impact is anticipated, it is prudent to be prepared and proactive into managing the associated risks. To that effect, stabilization of the amount and pattern of drinking, empowerment of the individual patient and the immediate environment to prepare and implement lifestyle changes in advance of stopping alcohol, and furthermore the avoidance, if possible, of detoxification by a gradual withdrawal might prevent or provide protection against or increase the ability of the patient and the immediate environment to cope with them.

There is some evidence that people who had more than two detoxifications do worse than those who had less than two detoxifications. Although some of the cognitive impairment observed might be pre-existing (i.e., as part of increasing vulnerability to addiction), this evidence indicates that there might be an accumulating effect with worsening of outcomes and reduction of the possibility of achieving sustainable outcomes. If this evidence is correct and the hypotheses that repeated detoxifications have a long-term negative impact, then it is crucial to avoid repetition of detoxifications and approach each detox as if it would be the last one. A proactive approach within the spirit of pre-habilitation to maximize the chances of lifelong abstinence following detoxification is even more relevant.

Further, evidence presented suggests that the medication used at the moment does not protect from or necessarily reverse the negative cognitive impact and therefore is not optimal to reduce the risk of relapse and possible long-term accumulative negative effects of detoxifications. Until such medication is developed, active participation with aftercare interventions to maintain abstinence or at least keep drinking at low risk level is crucial and every effort should be made for patients to continue their treatment beyond the end of detoxification. A pre-habilitation approach that exposes and familiarizes patients to psychosocial interventions will enhance their ability to participate in aftercare interventions.

There are several clinical questions for which we require evidence. How many detoxifications should we offer within a specific period of time? How soon after a relapse should we offer another detoxification? Is there a washout period following a detoxification or are these effects permanent? Does this mean that, following two failed detoxifications, there is no further negative impact and therefore detoxification should be offered at any given opportunity? Given the above clinical uncertainties and the potential risks indicated by the reviewed evidence and until further evidence provides answers, a new treatment paradigm based on the principles of pre-habilitation in addition to rehabilitation seems to have major advantages in providing aspects of the rehabilitation treatment before detoxification. SPADe provides such a model, in which a structured Cognitive Behavior Therapy-based intervention, which aims to stabilize drinking, introduce early lifestyle changes, and involve immediate social system into proactive changes to support the early stages of abstinence, is consistent with pre-habilitation and is supported by preliminary evidence that might be effective (11, 12). It is important though to remind ourselves that one of <br/>the primary objectives of a pre-habilitation treatment paradigm is the empowerment of the person with the drinking problem and for the immediate social environment to take responsibility for the problem and be active agents of the solution. Structured interventions prior to detoxification should be offered within the spirit of pre-habilitation and not as a screening process to manage the ever-reducing budgets for inpatient detoxification as suggested in the most recent report of PHE (10). If implemented to screen patients, then such a use of pre-detoxification groups could create barriers into accessing treatment and compromise rather than enhance long-term treatment outcomes (10).

## Author Contributions

CK: overall coordination of the manuscript with final editing and writing up of sections Introduction, Currently Recommended Treatment Paradigm to Manage Alcohol Detoxification, Learning and Habit Development in Humans, Examples of Implementation of Prehabilitation in Alcohol Dependence, and Conclusion. TD: written section Consequences of Repeated Detoxification of Patients Dependent on Alcohol; EP: written section Animal Models of Alcohol Withdrawal and Detoxification on Cognitive Impact; and AL-H: written section Brain Imaging of Alcohol Detoxification in Humans.

## Funding

The publication cost of this paper has been supported by the UK National Institute for Health Research (NIHR) under its Research for Patient Benefit (RfPB) Programme (grant reference number PB-PG-0815-20014).

## Conflict of Interest Statement

CK and TD declare that any personal research and the review paper was conducted in the absence of any commercial or financial relationships that could be construed as a potential conflict of interest. EP and AL-H declare that an unrestricted grant from Alcarelle funds a PhD studentship for EP (student) and AL-H (supervisor).
